# Submaximal low-load resistance exercise with blood flow restriction produces similar results to low-load exercise to failure for muscle size and strength, but not endurance

**DOI:** 10.1007/s00421-025-05949-1

**Published:** 2025-09-04

**Authors:** Ryo Kataoka, William B. Hammert, Yujiro Yamada, Robert W. Sallberg, Anna Kang, Jun Seob Song, Witalo Kassiano, Emily E. Metcalf, Jeremy P. Loenneke

**Affiliations:** 1 Department of Health, Exercise Science, and Recreation Management, Kevser Ermin Applied Physiology Laboratory, The University of Mississippi, University, P.O. Box 1848, Oxford, MS 38677 USA; 2 Department of Counseling, Health, and Kinesiology, Texas A&M University-San Antonio, San Antonio, TX USA; 3 Metabolism, Nutrition and Exercise Laboratory, State University of Londrina, Londrina, PR Brazil

**Keywords:** Resistance training, Kaatsu, Ischemia, Proximity to failure, Crossover effect

## Abstract

**Purpose:**

To examine the effects of submaximal low-load resistance exercise with and without blood flow restriction (BFR) on muscle size, strength, cross-education of strength, and muscular endurance with BFR compared to low-load exercise to failure.

**Methods:**

144 participants were randomly assigned to: (1) submaximal low-load exercise (LL, *n* = 37), (2) submaximal low-load exercise with BFR (LL + BFR, *n* = 35), (3) low-load exercise to failure (LL-Failure, *n* = 36), and (4) non-exercise control (CON, *n* = 36). Training consisted of 2 sets of 30% 1RM elbow flexion exercise, performed 3 days/week for 6 weeks. Repetitions performed by the submaximal groups were based on the muscular endurance test with BFR during pre-testing (70% of maximal BFR repetitions in week 1 and 95% in week 6).

**Results:**

LL + BFR led to greater increases in muscle thickness (0.14 cm) compared to LL (0.06 cm), and was comparable to LL-Failure (0.17 cm). 1RM strength gains were greater in LL (0.45 kg), LL + BFR (0.54 kg), and LL-Failure (0.34 kg) compared to CON (−0.36 kg), with no differences between training groups. There was no evidence of cross-education of strength. Changes in muscular endurance with BFR were greatest in LL-Failure (16.5 reps), followed by LL + BFR (10.0 reps), LL (4.2 reps), and CON (−0.03 reps).

**Conclusion:**

The effectiveness of BFR during submaximal exercise may depend on the specific adaptation targeted. Submaximal BFR produced muscle growth comparable to failure training. Neither BFR nor proximity to failure was necessary to maximize strength gains. Muscular endurance with BFR increased in all training groups, but improved the most with failure training.

**Supplementary Information:**

The online version contains supplementary material available at 10.1007/s00421-025-05949-1.

## Introduction

The addition of blood flow restriction to low-load resistance exercise has been shown to be an effective method for augmenting changes in muscle size and strength (Loenneke et al. 2012; Dankel et al. 2016). However, the independent effects of blood flow restriction remain less clear. Indeed, a central challenge in blood flow restriction research is determining how to prescribe the workload (i.e., load and repetitions) to isolate the effects of the blood flow restriction itself (Loenneke et al. 2025). Experimentally, if the goal is to test whether blood flow restriction is effective, the only difference between the two exercise groups should be the applied pressure (Hammert et al. 2024a). One common methodological approach is to prescribe the same number of sets and repetitions for all individuals [e.g., 30 repetitions followed by three sets of 15 repetitions at 30% of 1-repetition maximum (1RM)] (Laurentino et al. 2012; Neto et al. 2019; Yasuda et al. 2010; Davis et al. 2024). However, many participants are not able to achieve all of the prescribed repetitions under blood flow restriction (Mouser et al. 2017; Hughes and Patterson 2020; Norbury et al. 2024). This means that exercise conditions differ not only by the application of blood flow restriction, but also in the actual number of repetitions performed and proximity to task failure. The same limitation still persists even when failure is not reached, as the stimulus achieved is likely to vary depending on each individual’s muscular endurance capacity at a given relative load. These factors may partially explain the conflicting findings on muscle growth with repetition-matched protocols (Laurentino et al. 2012; Weatherholt et al. 2013).

Another methodological approach involves having one or both of the conditions exercise to task failure. Some studies have used a work-matched protocol, where the free-flow group matches the number of repetitions performed by the blood flow restriction group lifting to failure (Nielsen et al. 2012; Takarada et al. 2000, 2004; Kacin and Strazar 2011). This approach is problematic because it fails to account for how fatiguing the protocol is for the free-flow group. It also prevents the order from ever being randomized, which is an important component of experimental design (Senn 2013). An alternative strategy is to have all conditions exercise to failure (Dankel et al. 2017b). This controls for effort, but the differences in workload preclude conclusions being made about the effectiveness of blood flow restriction. Further, it has been suggested that the stimulus produced from exercising to failure might be so potent that it may overshadow the effects of blood flow restriction on certain variables (e.g., muscle growth) (Pignanelli et al. 2023; Hammert et al. 2023). As such, it becomes difficult to know whether the effects are due to blood flow restriction or lifting to failure.

Acute work from our laboratory found that applying blood flow restriction during submaximal low-load resistance exercise provided an additional stimulus for select markers of exercise-induced hypoalgesia (Hammert et al. 2024d). However, whether these findings would extend to chronic adaptations in skeletal muscle is unknown. Given the methodological limitations outlined above, the present study aimed to expand on acute data and examine the effects of submaximal low-load resistance exercise with blood flow restriction on three commonly measured outcomes in blood flow restriction research: muscle size, maximal strength, and local muscular endurance. Importantly, the present study accounted for proximity to failure under restriction. Building on previous work demonstrating an additive effect of blood flow restriction on contralateral strength gains during isometric exercise (Wong et al. 2024), we also explored whether blood flow restriction enhances the cross-education of strength in isotonic exercise. Lastly, these effects were compared to those in a group training to task failure, a condition often emphasized for maximizing muscular adaptations with low-load training (Weakley et al. 2023; Robinson et al. 2024; Flewwelling et al. 2025; Fisher et al. 2017). To our knowledge, no prior study has compared submaximal low-load resistance training with and without blood flow restriction to failure training without restriction.

## Methods

### Participants

A total of 186 healthy young untrained individuals were recruited for the project across the university campus. The sample size was determined based on our recent work that enabled us to detect differences between various resistance training groups in muscle size and strength (Bell et al. 2023; Wong et al. 2024; Song et al. 2024). The current study was part of a larger project that also examined whether the magnitude of skeletal muscle growth with resistance training is negatively influenced by the amount of muscle recruited within a given training period. Data from one group (elbow flexion + knee extension) was excluded, as that group was unrelated to the research question addressed in this paper. The final sample consisted of 144 participants (62 males and 82 females). Inclusion criteria were as follows: individuals (1) between the ages of 18 and 35 years; (2) not regularly engaging in resistance exercise or calisthenics within the past 6 months; (3) not having any orthopedic injuries preventing them from performing elbow flexion and knee extension exercises; (4) not used tobacco products regularly in the past 6 months; and (5) not taking any blood pressure medications. The participants were excluded if they met two or more of the following risk factors for thromboembolism (Motykie et al. 2000): body mass index ≥ 30; diagnosis of Crohn’s disease; varicose veins; family or personal history of deep vein thrombosis; or family or personal history of pulmonary embolism. These exclusions were precautionary and aligned with recommended practices in blood flow restriction research (Nascimento et al. 2022). All participants completed a physical activity readiness questionnaire (PAR-Q) and provided written informed consent prior to participating in the study, which was approved by the university’s institutional review board (Protocol #24–024).

### Study design

Participants had the pre-testing measurements taken in the following order: (1) height and body mass; (2) muscle thickness on the anterior portion of both upper arms; (3) arterial occlusion pressure (AOP); (4) 1RM strength for the unilateral elbow flexion exercise; and (5) local muscular endurance under restriction. Except for height and body mass, all of the tests were repeated at the post-testing visit. Following the pre-testing visit, participants were randomly assigned to one of four groups. Participants were stratified by sex and then randomly assigned to one of the following groups using RANDOM.ORG (https://www.random.org/): (1) submaximal low-load resistance exercise (LL); (2) submaximal low-load resistance exercise with blood flow restriction (LL + BFR); (3) low-load resistance exercise to failure (LL-Failure); and (4) a time-matched non-exercise control (CON). Participants in the training groups reported to the laboratory on 20 separate occasions (2 testing sessions), while the control group visited the laboratory only for the testing visits that matched the length of the training intervention (Hammert et al. 2024a). Researcher(s) supervised all training sessions in the laboratory, and participants trained three times a week for six weeks (18 training sessions) on non-consecutive days, with at least 24 h of rest between sessions. The post-testing visit occurred 2–5 days after the final training visit at a similar time of day as the pre-testing visit (± 1 h) to minimize any circadian influences on the measured variables. The participants were also asked to abstain from alcohol and exercise for 24 h, caffeine for 8 h, and eating for 2 h prior to the testing sessions.

### Muscle thickness

Muscle thickness was measured using B-mode ultrasound with a 4.2–13 MHz linear transducer (Logiq e, General Electric, Fairfield, CT) in a supine position. The right arm was measured first, followed by the left, with the measured arm slightly abducted (8.5 cm) and the palm facing upward. Straps were secured around the wrist, torso, and feet to minimize the movement. The ultrasound probe was coated with conductive gel and held lightly against the individual’s skin. Muscle thickness was captured using manufacturer-provided on-screen digital calipers and was defined as the distance from the muscle–bone interface to the muscle-subcutaneous fat interface, assessed to the nearest 0.01 cm. Measurements of the anterior upper arm were taken at 60% and 70% between the acromion process and the lateral epicondyle of the arm, corresponding approximately to the region overlying the biceps brachii. Three images were stored at each site on the ultrasound hard drive. Averaged values for each site were used for the subsequent analysis. The same researcher (RK) took all ultrasound images and completed all the image analyses in a blinded fashion following the data collection period for all participants.

### Arterial occlusion pressure (AOP)

The resting AOP was measured to apply a relative pressure for muscular endurance testing and the blood flow restriction training group. A 5-min seated rest period was provided before the pressure determination. The pressure was determined in a standing position where a 5-cm wide nylon pneumatic cuff (SC5 Hokanson Inc., Bellevue, WA) was placed at the most proximal portion of the dominant arm. An auditory signal of a pulse was found at the radial artery of the wrist using a Doppler probe (MD6 Hokanson Inc., Bellevue, WA). Once the Doppler probe could clearly detect a pulse, the cuff was slowly inflated (E20 Rapid Cuff Inflator Hokanson Inc., Bellevue, WA) from 50 mmHg until there was no detectable pulse. The pressure at which blood flow was no longer present was recorded as AOP (nearest mmHg), and the assigned percentage (80%) of this pressure was applied during muscular endurance testing and training. This process generally took less than 1 min per participant. The same individual (RK) placed the cuff and measured the AOP for pre- and post-testing visits.

### One-repetition maximum (1RM) strength test

1RM strength for the unilateral elbow flexion exercise was measured with load-adjustable dumbbells (measured to the nearest 0.22 kg). It was determined by finding the greatest load participants could lift one time with proper form through a full range of motion. 1RM measures were established in the dominant arm first, and then in the non-dominant arm. During each attempt, the participants were handed a loaded dumbbell and verbally encouraged to complete the full range of elbow flexion. A 90-s rest was given between attempts. The load was progressively increased until the participant was no longer able to lift a load greater than their previous heaviest successful attempt.

### Muscular endurance test (muscular endurance under restriction)

Muscular endurance under restriction was used as a performance outcome (Kacin and Strazar 2011; Hammert et al. 2024d) and to determine prescribed repetitions across sets for submaximal training groups. During pre-testing, participants completed a total of 3 sets of maximal repetitions on the unilateral bicep curl exercise at 30% of 1RM with the application of blood flow restriction (80% AOP), divided into two parts. Participants were instructed to complete repetitions at a cadence of 2 s per contraction (1 s concentric and 1 s eccentric muscle actions). The test was terminated if they could not keep pace with the metronome or could not lift the load through a full range of motion. In the first part, participants performed as many repetitions as possible for a single set. This set served as a performance outcome and to determine training repetitions for the first set of each training session. The cuff remained inflated throughout and was deflated immediately after the set. After a 5-min rest, the second part began. Participants completed two additional sets of bicep curl exercise under the same restricted pressure. During these sets, participants performed 70% of the repetitions completed on the first set (truncated to whole numbers), rested for 30 s, and then performed as many repetitions as possible on the second set. The first set was used to replicate the condition experienced during training before progressing to the second set (i.e., completing 70% of maximal blood flow restriction repetitions). The number achieved during the second set was used to determine the training repetitions for the second set during the intervention. The cuff remained inflated throughout the two sets of exercise and was deflated immediately after the conclusion of the second set. During post-testing, participants performed a single set (i.e., using 30% of pre-intervention 1RM) of maximal repetitions as a performance outcome to compare changes between groups following the intervention. The applied pressure was based on the resting AOP from the pre-testing visit (80% AOP).

### Training protocol

Training sessions were carried out three times per week on non-consecutive days for six weeks (18 training sessions). All training groups trained their dominant arm. The dominant arm was defined as the preferred arm for throwing a ball. The LL and LL + BFR groups involved the submaximal protocol in bicep curl exercise for two sets with or without blood flow restriction. The prescribed repetitions across sessions were based on the muscular endurance performance of the first and second sets during the pre-testing. The initial training week used 70% of the maximum repetitions performed with blood flow restriction, and the number of repetitions progressed by 5% each week (i.e., every three training sessions) to ensure participants continued to train at a submaximal intensity. Repetitions performed were truncated to whole numbers. By the end of training (week 6), participants performed 95% of their pre-testing maximal repetitions for two sets. Each set was separated by a 30-s rest period. Both prescribed repetitions and completed repetitions were recorded throughout the training visits. The only difference between the LL and LL + BFR groups was that the LL + BFR group applied blood flow restriction at 80% of AOP, while the LL group still matched for a percentage of maximal blood flow restriction repetitions. The cuff remained inflated throughout the entire training session and deflated immediately after the conclusion of the second set. The LL-Failure group performed two sets of biceps curl exercise to failure each session. All repetitions were performed at a cadence of 2 s per contraction (1 s concentric and 1 s eccentric muscle actions). The prescribed load was set at 30% 1RM. The initial load was maintained throughout the intervention for all training groups; therefore, the progression occurred through performing more repetitions in each set.

### Statistical analysis

Changes in muscle size, strength, and muscular endurance under restriction (pre- to post-measurements) were compared using Bayes factors for informative hypotheses (BAIN) in JASP version 0.19.3. The ANCOVA function of BAIN was used with the pre-values serving as the covariate. BAIN uses a fraction of the information in the data to specify the variance of the prior distribution (Hoijtink et al. 2019). Based on our theory-driven expectations about the likely direction and magnitude of effects, BAIN enables direct comparison of multiple hypotheses by quantifying how much more likely one hypothesis is than another (given the observed data). Specific hypotheses were determined a priori and evaluated by comparing Bayes factors (BF) and the posterior probabilities between models. Hypotheses tested are found in Table 1. We also conducted a post-hoc mediation analysis to examine if changes in strength were mediated by a change in muscle size and further whether a change in local muscular endurance was mediated by a change in muscle strength (Supplementary Fig. 1 and 2).

**Table 1:** Description of the hypotheses (H) compared for each variable. H1, hypothesis 1; H2, hypothesis 2; H3, hypothesis 3

Variables		Hypothesis compared
Muscle thickness		
LL vs. LL + BFR vs. LL-failure vs. CON	H1	LL + BFR = LL-failure > LL > CON
	H2	LL-Failure > LL + BFR > LL > CON
	H3	LL-Failure > LL + BFR = LL = CON
1RM strength (trained arm)		
LL vs. LL + BFR vs. LL-failure vs. CON	H1	LL + BFR = LL-failure > LL > CON
	H2	LL + BFR = LL-failure = LL > CON
	H3	LL-Failure > LL + BFR > LL > CON
1RM strength (untrained arm)		
LL vs. LL + BFR vs. LL-failure vs. CON	H1	LL + BFR > LL-failure = LL = CON
	H2	LL + BFR > LL-failure > LL = CON
	H3	LL + BFR = LL-failure = LL = CON
Muscular endurance under restriction		
LL vs. LL + BFR vs. LL-failure vs. CON	H1	LL + BFR > LL-failure > LL > CON
	H2	LL + BFR = LL-failure > LL > CON
	H3	LL-failure > LL + BFR > LL > CON

*LL* submaximal low-load resistance exercise, *LL + BFR* submaximal low-load resistance exercise with blood flow restriction, *LL-failure* low-load resistance exercise to failure, *CON* time-matched non-exercise control group

## Results

### Demographic

A total of 149 participants were randomly assigned to four groups. Four participants withdrew from the study due to the following reasons: one = loss of interest, one = breakup with their significant other, one = start of resistance training outside of study, and one = work schedule. One participant was excluded from the statistical analysis due to engaging in the heavy preacher curl exercise outside of the study. Thus, the final sample consisted of 144 participants (LL: *n* = 37, LL + BFR: *n* = 35, LL-Failure: *n* = 36, CON: *n* = 36). The participant demographics are presented in Table 2, and the pre-, post-, and change score values for the primary outcomes can be found in Table 3. All participants in the submaximal groups were able to complete the prescribed repetitions. One participant missed one training session. No adverse responses to training were observed or reported. Individual changes for the main outcomes are found in Supplementary Fig. 3 and Fig. 4. During the review process, we added Supplementary Fig. 5 which visually depicts the unadjusted change scores for each group, separated by sex.

**Table 2:** Participant demographics separated by group

Variable	LL (*n* = 37)	LL + BFR (*n* = 35)	LL-failure (*n* = 36)	CON (*n* = 36)
Sex (male/female)	16/21	15/20	16/20	15/21
Age (years)	21.4 (3.8)	20.8 (2.9)	20.1 (2.7)	21.2 (3.7)
Body mass (kg)	67.7 (15.7)	67.5 (19.2)	70.1 (18.0)	72.6 (22.2)
Height (cm)	168.5 (8.8)	170.0 (9.0)	170.8 (9.3)	169.9 (10.0)
AOP (mmHg)	141.2 (20.8)	139.3 (17.9)	143.6 (20.3)	142.7 (21.6)

Mean (SD) age, body mass, height, and arterial occlusion pressure at the baseline

*n* sample size, *kg* kilograms, *cm* centimeters, *AOP* arterial occlusion pressure, *mmHg* millimeters of mercury, *SD* standard deviation

**Table 3:** The mean and standard deviation (SD) for pre-, post-, and change score values separated by group (LL: *n* = 37, LL + BFR: *n* = 35, LL-failure: *n* = 36, CON: *n* = 36)

	Pre (SD)	Post (SD)	Change (SD)
Muscle thickness (60%) cm			
LL	2.62 (0.50)	2.68 (0.51)	0.06 (0.11)
LL + BFR	2.55 (0.67)	2.70 (0.69)	0.15 (0.10)
LL-failure	2.67 (0.47)	2.85 (0.51)	0.18 (0.14)
CON	2.71 (0.65)	2.69 (0.64)	-0.02 (0.10)
Muscle thickness (70%) cm			
LL	2.99 (0.52)	3.06 (0.54)	0.07 (0.12)
LL + BFR	2.90 (0.68)	3.03 (0.68)	0.13 (0.12)
LL-Failure	3.05 (0.49)	3.22 (0.53)	0.17 (0.12)
CON	3.06 (0.68)	3.01 (0.67)	-0.05 (0.12)
1RM strength (trained arm) kg			
LL	12.46 (4.27)	12.93 (4.67)	0.46 (0.89)
LL + BFR	12.69 (5.25)	13.24 (5.29)	0.55 (0.80)
LL-failure	12.78 (3.67)	13.13 (3.75)	0.34 (1.3)
CON	13.32 (6.10)	12.94 (5.46)	-0.37 (0.90)
1RM strength (untrained arm) kg			
LL	11.84 (3.66)	12.02 (3.99)	0.17 (0.87)
LL + BFR	12.00 (5.09)	12.29 (5.15)	0.29 (0.79)
LL-failure	12.20 (3.79)	12.51 (3.84)	0.30 (1.4)
CON	12.40 (5.69)	12.62 (5.72)	0.22 (0.87)
Muscular endurance under restriction (rep)			
LL	29.13 (5.85)	33.37 (7.28)	4.24 (5.43)
LL + BFR	27.77 (5.20)	37.80 (11.36)	10.02 (9.71)
LL-failure	28.13 (6.24)	44.72 (23.01)	16.58 (22.09)
CON	29.25 (7.14)	29.22 (8.53)	-0.02 (4.39)

### Changes in muscle thickness

**Fig. 1:**
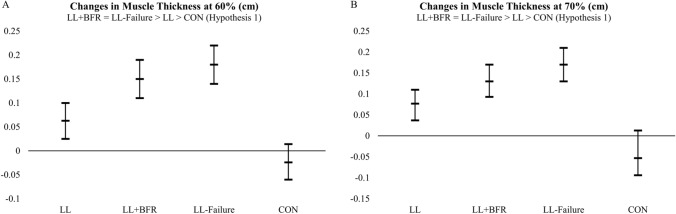
The adjusted (covaried for baseline) changes in muscle thickness on the anterior upper arm (cm) at the 60% (**A**) and 70% (**B**) sites in the dominant arm. The mean changes in muscle thickness are represented by the middle bar, and the upper and lower bars represent the 95% credible intervals. The hypotheses at the top of each figure represent the hypothesis with the greatest posterior probability. LL, submaximal low-load resistance exercise; LL + BFR, submaximal low-load resistance exercise with blood flow restriction; LL-Failure, low-load resistance exercise to failure; CON, time-matched non-exercise control group

Of the hypotheses compared (Table 1), the posterior probability favored the first hypothesis (H1) for the changes in muscle thickness at 60% and 70% sites. This provides evidence that changes in muscle thickness at the 60% site were greater in LL + BFR (0.15 cm) and LL-Failure (0.18 cm) compared to LL (0.06 cm) and CON (−0.02 cm). The changes in muscle thickness were comparable between LL + BFR and LL-Failure, while the changes in muscle thickness were greater in LL compared to CON (Fig. 1A; Table 3). Similarly, changes in muscle thickness at the 70% site were greater in LL + BFR (0.13 cm) and LL-Failure (0.17 cm) compared to LL (0.07 cm) and CON (−0.05 cm). The changes in muscle thickness were comparable between LL + BFR and LL-Failure, while the changes in muscle thickness were greater in LL compared to CON (Fig. 1B, Table 3). The posterior probabilities for each hypothesis (including the unconstrained model, i.e., HU) are as follows: H1: 0.54; H2: 0.43; H3: 7.97 × 10^–9^; HU: 0.02 for the 60% site; and H1: 0.49; H2: 0.47; H3: 9.01 × 10^–10^; HU: 0.02 for the 70% site. Additionally, there was no evidence of muscle growth in the untrained arm (data not shown).

### Changes in 1RM strength

**Fig. 2:**
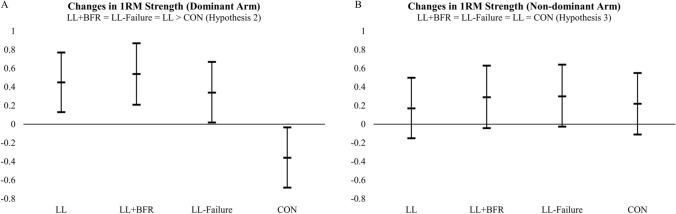
The adjusted (covaried for baseline) changes in 1RM strength (kg) in the dominant (**A**) and non-dominant (**B**) arms. The mean changes in strength are represented by the middle bar, and the upper and lower bars represent the 95% credible intervals. The hypotheses at the top of each figure represent the hypothesis with the greatest posterior probability. LL, submaximal low-load resistance exercise; LL + BFR, submaximal low-load resistance exercise with blood flow restriction; LL-Failure, low-load resistance exercise to failure; CON, time-matched non-exercise control group

Of the hypotheses compared (Table 1), the posterior probability favored the second hypothesis (H2). This provides evidence that changes in 1RM strength in the trained arm were greater in LL + BFR (0.54 kg), LL-Failure (0.34 kg), and LL (0.45 kg) compared to CON (−0.36 kg). However, there were no differences observed between training groups (Fig. 2A; Table 3). For the 1RM strength changes in the untrained arm, of the hypotheses compared (Table 1), the posterior probability favored the third hypothesis (H3). This provides evidence that changes in 1RM strength in the untrained arm were not different between LL + BFR (0.29 kg), LL-Failure (0.30 kg), LL (0.17 kg), and CON (0.22 kg) (Fig. 2B; Table 3). The posterior probabilities for each hypothesis (including the unconstrained model, i.e., HU) are as follows: H1: 0.17; H2: 0.79; H3: 0.01; HU: 0.01 for the trained arm; and H1: 0.15; H2: 0.03; H3: 0.8; HU: 0.002 for the untrained arm. There was no evidence of mediation for the relative indirect effects comparing training groups to the control group for 1RM strength (trained arm) changes through muscle growth (Supplementary Table 1).

### Changes in muscular endurance under restriction

**Fig. 3:**
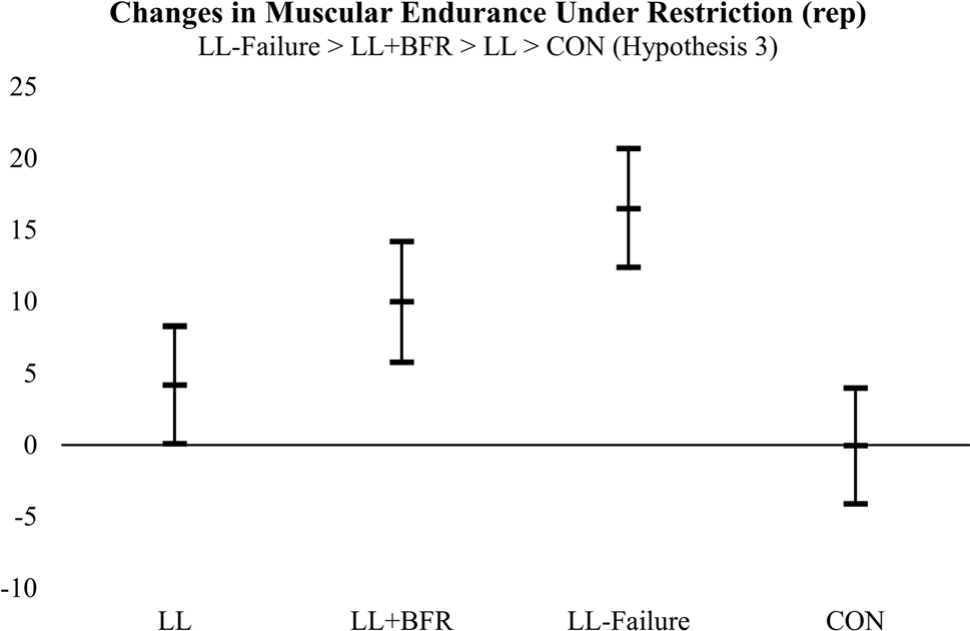
The adjusted (covaried for baseline) changes in muscular endurance under restriction (rep). The mean changes in muscular endurance under restriction are represented by the middle bar, and the upper and lower bars represent the 95% credible intervals. The hypotheses at the top of each figure represent the hypothesis with the greatest posterior probability. LL, submaximal low-load resistance exercise; LL + BFR, submaximal low-load resistance exercise with blood flow restriction; LL-Failure, low-load resistance exercise to failure; CON, time-matched non-exercise control group

Of the hypotheses compared (Table 1), the posterior probability favored the third hypothesis (H3). This provides evidence that changes in muscular endurance under restriction were greatest in LL-Failure (16.5 reps), followed by LL + BFR (10.0 reps), LL (4.2 reps), and CON (-0.03 reps) (Fig. 3, Table 3). The posterior probabilities for each hypothesis (including the unconstrained model, i.e., HU) are as follows: H1: 0.01; H2: 0.14; H3: 0.80; HU: 0.03. There was no evidence of serial mediation (exercise → muscle growth → changes in strength → changes in muscular endurance) for the relative indirect effects comparing training groups to the control group for local muscular endurance (Supplementary Table 2).

## Discussion

Previous studies have been unable to draw definitive conclusions on the independent effects of blood flow restriction during low-load resistance exercise due to difficulties prescribing the workload. Here, we accounted for proximity to failure under restriction and showed that: (1) submaximal low-load resistance exercise with blood flow restriction augmented muscle growth compared to the same exercise without blood flow restriction; (2) muscle growth with blood flow restriction was comparable to that observed in the group training to failure; (3) the application of blood flow restriction and exercising to failure had no greater effects on changes in 1RM and the cross-education of strength than did submaximal low-load exercise without blood flow restriction; (4) training to failure led to greater changes in muscular endurance under restriction than submaximal exercise with and without blood flow restriction. Taken together, the data suggest that the effectiveness of blood flow restriction during submaximal exercise depends on the specific adaptation targeted.

### Muscle growth

Training to or near failure leads to high levels of muscle activation (Morton et al. 2019), which is often considered necessary to maximize muscle growth with low-load resistance exercise (Weakley et al. 2023; Robinson et al. 2024; Fisher et al. 2017; Flewwelling et al. 2025). Our study demonstrated that training to failure resulted in greater muscle growth compared to submaximal training without blood flow restriction. When blood flow restriction was applied to submaximal exercise, muscle growth became more comparable to that of a group trained to failure. However, results from our BAIN analysis found a split in the posterior probabilities between the hypothesis that blood flow restriction training was similar to failure training and the hypothesis that failure training would be greater than blood flow restriction training for muscle growth. Thus, more evidence is needed to know if training to task failure could offer a marginal advantage for muscle growth. Nonetheless, comparable results were achieved despite substantially lower volume progression in the blood flow restriction group [i.e., LL + BFR: 8.6 vs. LL-failure: 239 repetitions (the average number of repetitions increased per session over 6 weeks)], highlighting that volume progression may not necessarily be a major driver of early muscle growth (Hammert et al. 2024b). While our study did not measure any molecular markers to elucidate the mechanisms underlying muscle growth, the mechanisms triggered by blood flow restriction are likely similar to those of traditional resistance exercise at the muscle fiber level once a sufficient stimulus is achieved (Ellefsen et al. 2015). The efficacy of blood flow restriction may stem from the accumulation of metabolites within the exercising muscles to augment fatigue-induced muscle activation (Dankel et al. 2017a). This stimulation is likely accompanied by an increased rate of muscle protein synthesis through the phosphorylation of associated signaling cascades (e.g., mTORC1) (Fujita et al. 2007; Fry et al. 2010), which may play a key role in promoting muscle growth at a submaximal intensity. Although our study did not include a blood flow restriction group that trained to failure, previous studies found that non-failure exercise with blood flow restriction increased muscle size to a similar extent as training to failure with blood flow restriction (Sieljacks et al. 2019). Additionally, it appears that blood flow restriction does not appreciably enhance muscle growth beyond what is achieved through failure training (Fahs et al. 2015; Farup et al. 2015; Pignanelli et al. 2020). Thus, the utility of blood flow restriction may be most impactful for muscle growth when paired with submaximal resistance exercise.

While some previous studies have reported greater muscle growth with blood flow restricted exercise compared to similar protocols without blood flow restriction (Loenneke et al. 2012; Dankel et al. 2016), other investigations using comparable protocols have produced conflicting results (Laurentino et al. 2012; Weatherholt et al. 2013). These discrepancies may arise from not accounting for individual differences in local muscular endurance under restriction (e.g., prescribing the same number of sets and repetitions for all individuals). Our protocol ensured that all individuals in the submaximal groups exercised at a *submaximal* intensity (i.e., not to task failure) by matching for a percentage of maximal blood flow restriction repetitions. This approach allowed us to better isolate the effects of blood flow restriction at a submaximal intensity. It is also notable that the submaximal training groups never surpassed the maximal repetition capacity achieved in the untrained state. Given that the prescribed load remained constant throughout the intervention, individuals likely trained at an even lower proximity to failure over time as their muscular endurance increased (discussed below). Future studies could examine whether the muscle growth with a submaximal blood flow restriction protocol would exceed that of a condition matched for proximity to failure without blood flow restriction (i.e., prescribing the same percentage of repetition to failure without blood flow restriction).

### Maximal strength

Low-load resistance exercise in the trained limb increased 1RM strength compared to the control group, with no differences between training groups. Our results suggest that increases in 1RM strength were not dependent on blood flow restriction. This is consistent with previous work that employed a constant load throughout training (Buckner et al. 2020; Jessee et al. 2018; Davis et al. 2024). It also appears that strength gains were not dependent on proximity to failure. Since the prescribed repetitions for the submaximal groups were based on the maximal endurance capacity under restriction, the submaximal exercise group without blood flow restriction likely performed well below their maximal achievable repetitions during training. Similar findings were echoed in Sieljacks et al. (2019), who showed similar strength gains when participants trained both legs under blood flow restriction, with one leg exercising to failure and the other completing 75% of those repetitions. The lack of additive effects from blood flow restriction or training to failure may also make sense from the perspective of motor skill acquisition, as these training protocols may not sufficiently replicate the demands of the strength test to develop the skills to lift a heavy load. Further, although muscle growth is often considered a candidate mechanism for strength gains, we found no evidence that growth mediated the change in muscle strength, which is in alignment with previous findings (Jessee et al. 2021; Spitz et al. 2023; Wong et al. 2025).

Our results may seem at odds with some previous findings reporting enhanced strength gains with blood flow restriction (Loenneke et al. 2012; Dankel et al. 2016). It is difficult to reconcile the results of our study; however, the interpretation of those findings may be confounded by factors that may influence strength adaptations. For example, some studies performed repeated maximal strength testing throughout the training intervention to update the training load (Weatherholt et al. 2013; Colomer-Poveda et al. 2017; Shinohara et al. 1998; Sousa et al. 2017; Pignanelli et al. 2020; Burgomaster et al. 2003), which may influence strength adaptations (Spitz et al. 2020). Others used within-subject designs (Takarada et al. 2000; Lixandrão et al. 2015; Counts et al. 2016; Moore et al. 2004) that risk strength transfer between limbs via cross-education of strength (Bell et al. 2023) or only reported maximal strength in movements that were not directly trained (i.e., trained isotonically but tested isokinetically and isometrically) (Takarada et al. 2004, 2002). These points should be considered when comparing our results to previous findings. However, we acknowledge that some studies have reported augmented strength gains with blood flow restriction even in the absence of those potential confounding factors (Wong et al. 2024; Laurentino et al. 2012). Future research should clarify when blood flow restriction may enhance strength adaptations.

### Cross-education of strength

We hypothesized that applying blood flow restriction would augment the cross-education of strength, as seen in prior studies with isometric resistance training (Wong et al. 2024). However, cross-education of strength was not observed in any of the training groups. The reason for this discrepancy is unclear; however, with isotonic training, the stimulus necessary to induce cross-education of strength may be load dependent (Colomer-Poveda et al. 2021; Bell et al. 2023; Dankel et al. 2020; Song et al. 2024). Colomer-Poveda et al. (2021) found that strength gains in the untrained limb occurred only in the high-load training group (75% vs. 25% 1RM), and lifting to failure at 75% 1RM did not enhance this response. While some have proposed that the magnitude of strength changes in the trained limb may influence the strength changes in the untrained limb (Manca et al. 2017), this relationship is likely multifactorial and not solely dependent on the magnitude of strength changes of the trained side (Wong et al. 2024). In our study, the use of a low-load protocol (30% 1RM) or a difference in exercise volume (e.g., two sets in ours vs. four sets in Wong et al. (2024)) may have contributed to the absence of cross-education effects in the untrained limb.

### Muscular endurance under restriction

Several studies have examined the effects of blood flow restriction training on local muscle endurance (Hammert et al. 2024c, 2025), but only one study has evaluated changes in muscular endurance under restriction (Kacin and Strazar 2011). We found that submaximal resistance exercise with blood flow restriction augmented the changes in muscular endurance under restriction relative to the same exercise without blood flow restriction, and the greatest improvement was observed in the group training to failure. Notably, even the submaximal group without blood flow restriction, whose training was least specific to the endurance test, still demonstrated improvement. This agrees with previous research from Martorelli et al. (2017), as well as a review of the literature that concluded absolute muscular endurance is capable of increasing through various training protocols (Hammert et al. 2025). Testing muscular endurance with blood flow restriction intensifies physiological demands, such as reduced oxygen delivery (Petrick et al. 2019), metabolite accumulation (Suga et al. 2012), and exercise-induced discomfort (Dankel et al. 2019). As such, improvements under these testing conditions may serve as a proxy for enhanced physiological tolerance to those stressors. The larger gains observed in the submaximal blood flow restriction and failure groups may be attributed to adaptations such as enhanced angiogenic gene expression and capillarization (Ferguson et al. 2018; Larkin et al. 2012), as well as improved tolerance to discomfort (Mattocks et al. 2019). Several studies have shown that low-load resistance training to failure, with and without blood flow restriction, elicits similar improvements in muscular endurance (Buckner et al. 2020; Fahs et al. 2015). However, Jessee et al. (2018) reported that very low-load training with 80% AOP to failure (at some point across the four sets) further enhanced muscle endurance relative to other failure-based protocols. Those findings may suggest that while training to failure alone may largely saturate the adaptations for muscular endurance, blood flow restriction, under certain conditions, may exert an additive effect even when training to failure.

Our serial mediation analysis indicated that increases in muscular endurance under restriction were not explained by a sequential pathway of muscle growth leading to strength gains. This may be related to the relatively smaller increases in 1RM with lower load protocols. Previous work has shown that differences in muscular endurance between high-load and low-load training are mediated through differences in maximal strength (Chatlaong et al. 2022). Future research could compare endurance testing with or without blood flow restriction across failure and submaximal training groups with or without blood flow restriction to clarify whether training to failure, blood flow restriction, or their interaction plays a greater role in improving local muscular endurance.

## Limitations

Our statistical model assumes equal initial probabilities for all hypotheses. While each hypothesis tested was considered physiologically plausible, their prior odds were unlikely to be truly equal. We elected to use this approach because we had specific hypotheses we aimed to compare head-to-head. Muscle thickness was measured using B-mode ultrasound, which is not considered the gold standard. However, prior research provided similar conclusions on resistance training-induced muscle growth, whether measured with ultrasound or MRI (Franchi et al. 2018; Loenneke et al. 2019). The current study maintained a constant exercise load throughout the training intervention. While this may be viewed as a limitation, we view it as a strength in that it enabled us to better discern whether any adaptations were due to blood flow restriction or overload from the resistance training. Previous high-load training studies have shown that progressing training loads may not always lead to greater muscle strength gains within short time frames (Plotkin et al. 2022; Chaves et al. 2024; Hammert et al. 2023). Whether a similar effect occurs with low-load training is not known but could be explored further. While our methodology accounted for each individual’s local muscular endurance capabilities under restriction, it is possible that the same relative percentage still scaled differently on a physiological level. However, this limitation likely applies to various forms of submaximal protocols. Finally, it is unknown whether the results can be generalized to different muscle groups.

## Conclusion

Our methodology differed from previous studies, which prescribed the same number of repetitions for all individuals, irrespective of endurance capacity. By prescribing the repetitions relative to an individual’s maximal endurance capacity under restriction, we offered insights into the effects of blood flow restriction on submaximal resistance exercise. We found that blood flow restriction enhanced muscle growth when resistance exercise is performed at a submaximal intensity. Notably, increases in muscle size were comparable to those in the group training to task failure. We also demonstrated that 1RM strength increases in the trained arm were not dependent on blood flow restriction or the proximity to failure, suggesting that strength adaptations can occur under various submaximal conditions. However, there was no evidence of cross-education of strength in the untrained arm. Lastly, the application of blood flow restriction at a submaximal intensity augmented muscular endurance under restriction compared to the same exercise without blood flow restriction, and greater adaptations occurred for those who trained more similarly to how testing was performed (i.e., task failure under restriction).

## Supplementary Information

Below is the link to the electronic supplementary material.

Supplementary file1 (PDF 488 KB)

## Data Availability

All data are available upon reasonable request.

## Abbreviations

1RM: 1-Repetition maximum
AOP: Arterial occlusion pressure
BAIN: Bayes factors for informative hypotheses
BF: Bayes factor
BFR: Blood flow restriction
CON: Control
LL: Low-load exercise
MRI: Magnetic resonance imaging
PAR-Q: Physical activity readiness questionnaire
