# Supplementary material for: Submaximal low-load resistance exercise with blood flow restriction produces similar results to low-load exercise to failure for muscle size and strength, but not endurance
Source: Eur J Appl Physiol. 2025 Sep 4;126(2):825–37. doi: 10.1007/s00421-025-05949-1 (PMC12948889; doi:10.1007/s00421-025-05949-1)
Supplement: Supplementary file 1 — Supplementary file1 (PDF 488 KB) [file 421_2025_5949_MOESM1_ESM.pdf]

Statistical analyses

First, to evaluate the effects of training on changes in 1RM strength, with changes in muscle thickness as mediator, a simple mediation model was constructed. Second, we constructed a serial mediation analysis to evaluate whether changes in muscle thickness in response to training mediate changes in 1RM strength, which in turn mediate changes in local muscular endurance. Both analyses were performed using the PROCESS 4.2 macro for SPSS. To account for their possible influences on an individual's potential change, sex and pre-training values of muscle thickness and 1RM strength were included as covariates in the simple mediation model. Pre-testing values of muscular endurance were additionally included as a covariate in the serial mediation model. All coefficients for between-group comparisons are provided relative to the control group. For the relative indirect effects, percentile bootstrap confidence intervals were constructed with 5000 draws. All data is reported as the regression coefficient (95% confidence interval). Statistical significance was set at $p < 0.05$.

Figure legend

Supplementary Figure 1. Simple mediation model. Conceptual diagram detailing the conceptual framework for assessing the direct and indirect effects of training protocols (each compared to Control) on changes in 1RM strength through changes in muscle thickness.

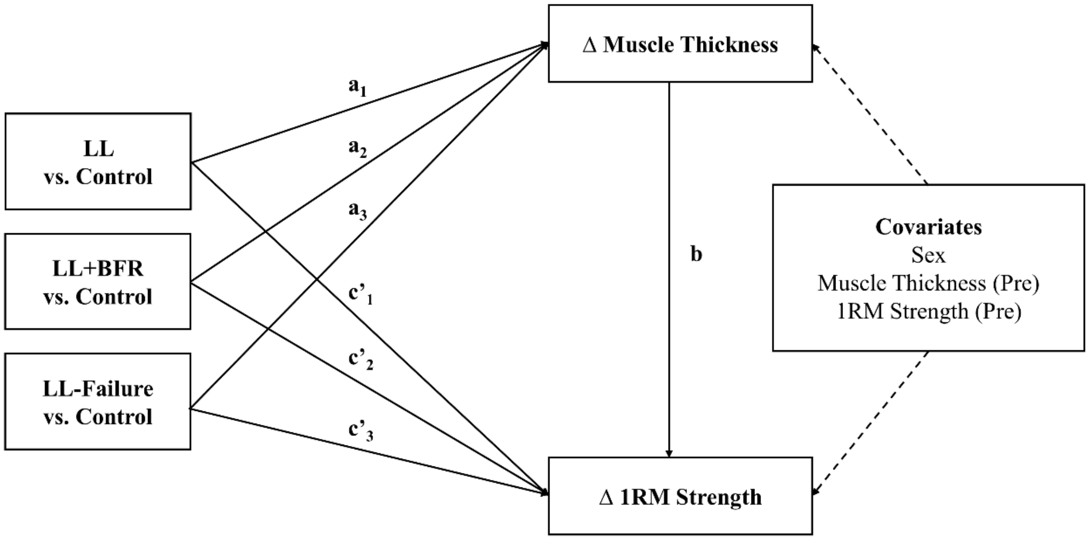

Supplementary Figure 2. Serial mediation model. Conceptual diagram detailing the conceptual framework for assessing the direct and indirect effects of training protocols (each compared to Control) on changes in 1RM strength through changes in muscle thickness and changes in 1RM strength, alone and in series.

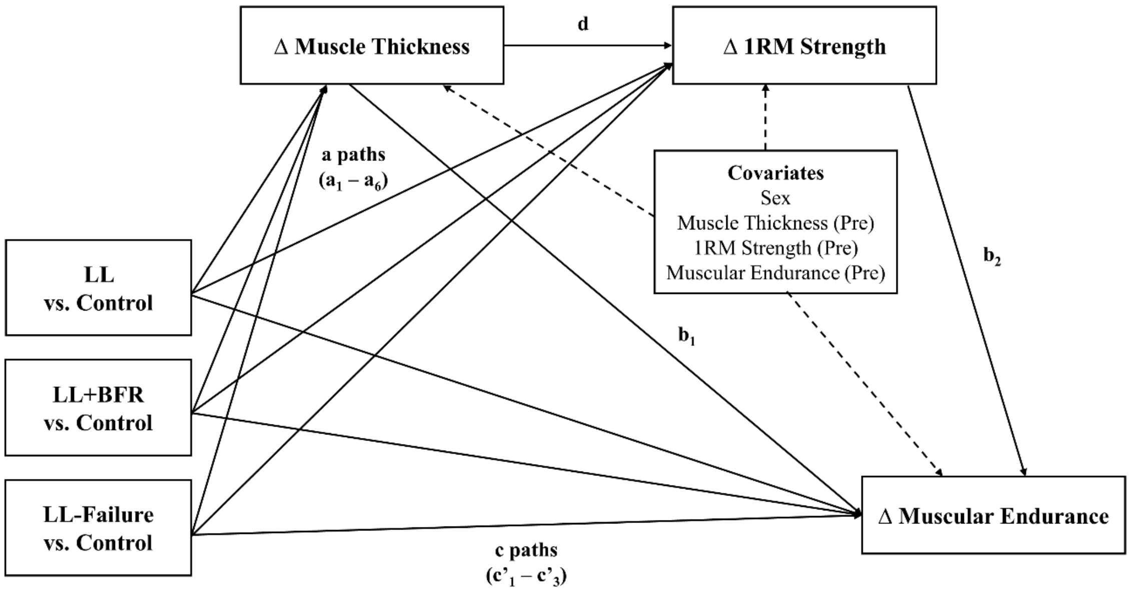

Supplementary Figure 3. Change in Dominant Arm muscle thickness at 60% and 70% site.

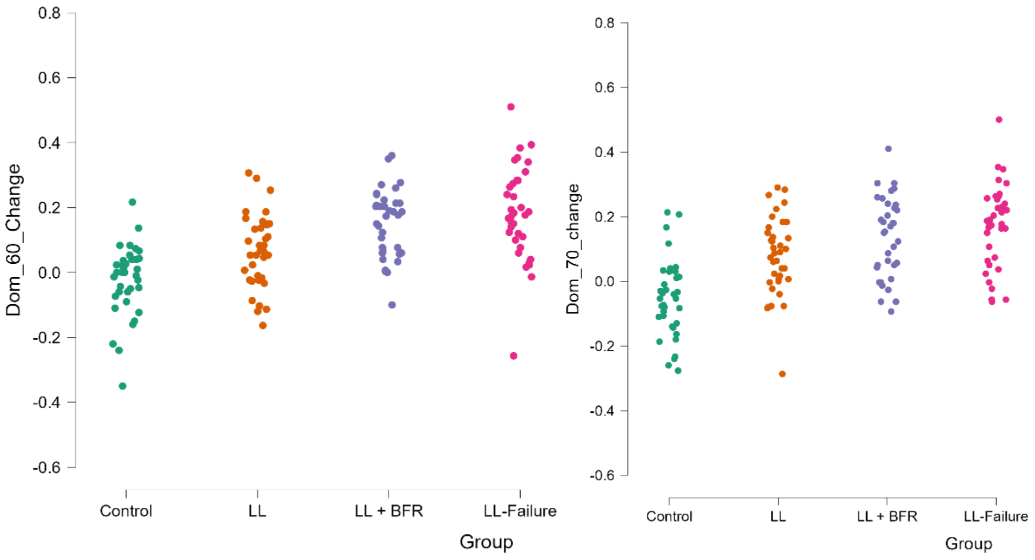

Supplementary Figure 4. Change in Dominant Arm Strength (1RM) and repetitions under blood flow restriction.

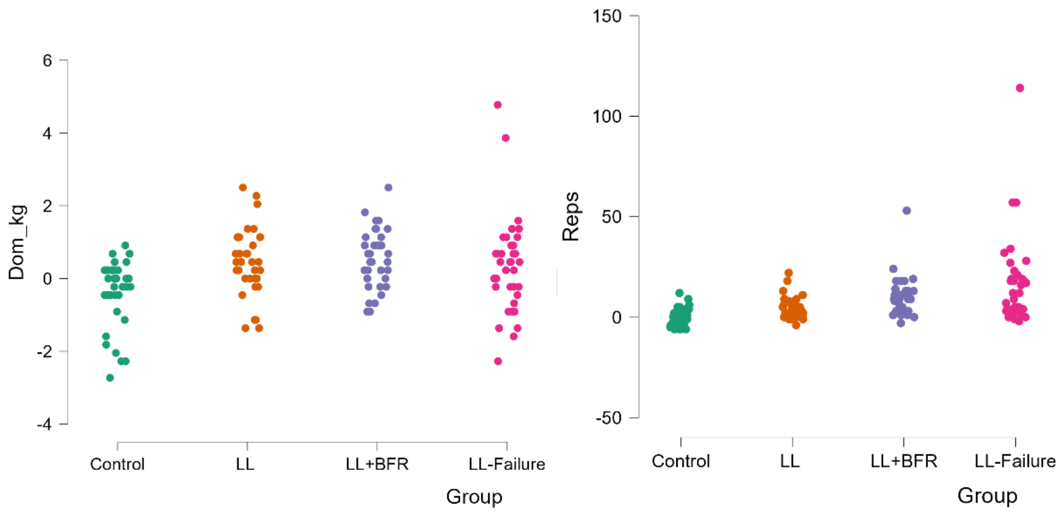

Supplementary Figure 5. Unadjusted chnages in (A) Dominant Arm Strength (1RM), (B) Non-dominant Arm Strength, (C) average muscle thickness, and (D) repetitions under blood flow restriction. Male are coded as 0 and females are coded as 1.

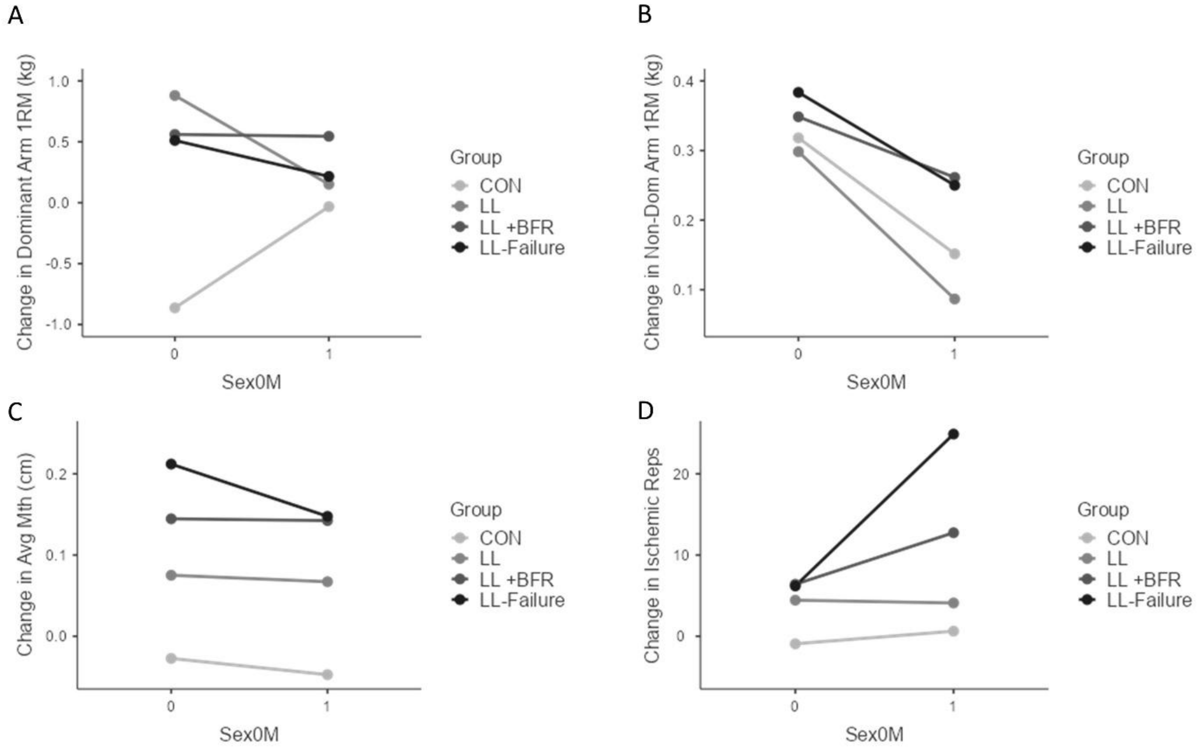

Supplementary Table 1. Estimated regression coefficient: Simple mediation model

	R^2	Predictor	Path	Coefficient	95% CI	*p* value
**Δ Muscle Thickness (cm)**	0.377					
		LL	a_1	0.108	0.056, 0.159	< 0.001
		LL+BFR	a_2	0.177	0.124, 0.230	< 0.001
		LL-Failure	a_3	0.214	0.163, 0.266	< 0.001
		constant	i_M	0.057	-0.104, 0.218	0.483
**Δ 1RM Strength (kg)**	0.248					
		LL	c'_1	0.596	0.127, 1.064	0.013
		LL+BFR	c'_2	0.666	0.147, 1.185	0.012
		LL-Failure	c'_3	0.315	-0.226, 0.857	0.252
		Δ Muscle Thickness	b	1.533	0.084, 2.983	0.038
		constant	i_Y	-0.060	-1.446, 1.326	0.931
**Indirect Effects (Simple Mediation)**						
			a_1*b	0.165	-0.014, 0.384	
			a_2*b	0.272	-0.024, 0.587	
			a_3*b	0.329	-0.030, 0.715	

Estimated regression coefficients for the simple mediation model (1RM strength). $a_{1\text{-}3}$ = adjusted mean difference in muscle thickness (Δ muscle thickness) between each training group and Control, $c'_{1\text{-}3}$ = adjusted relative direct effect of each training group compared to Control, b = adjusted effect of changes in muscle thickness on changes in 1RM strength, $a_{1\text{-}3}$*b = adjusted relative indirect effect of each training group on changes in 1RM strength through changes in muscle thickness compared to Control. Sex and pre-training values of muscle thickness and 1RM strength were included as covariates.

Supplementary Table 2. Estimated regression coefficient: Serial mediation model

	R^2	Predictor	Path	Coefficient	95% CI	*p* value
**Δ Muscle Thickness (cm)**	0.378					
		LL	a_1	0.107	0.056, 0.159	< 0.001
		LL-BFR	a_2	0.176	0.124, 0.229	< 0.001
		LL-Failure	a_3	0.213	0.161, 0.266	< 0.001
		constant	i_{M1}	0.071	-0.112, 0.254	0.444
**Δ 1RM Strength (kg)**	0.248					
		LL	a_4	0.596	0.126, 1.066	0.013
		LL-BFR	a_5	0.668	0.146, 1.189	0.012
		LL-Failure	a_6	0.317	-0.227, 0.861	0.251
		Δ Muscle Thickness	d	1.536	0.081, 2.991	0.038
		constant	i_{M2}	-0.101	-1.676, 1.473	0.898
**Δ Muscular Endurance (rep)**	0.292					
		LL	c'_1	3.727	-2.384, 9.839	0.229
		LL-BFR	c'_2	9.665	2.883, 16.447	0.005
		LL-Failure	c'_3	17.749	10.794, 24.704	< 0.001
		Δ Muscle Thickness	b_1	-10.910	-29.692, 7.871	0.252
		Δ 1RM	b_2			
		constant	i_Y	9.673	-10.341, 29.687	0.340
**Indirect Effects (Serial Mediation)**						
Mediator: Δ Muscle Thickness						
			$a_1{*}b_1$	-1.177	-3.376, 0.363	
			$a_2{*}b_1$	-1.929	-5.176, 0.587	
			$a_3{*}b_1$	-2.334	-6.400, 0.655	

Mediator: Δ 1RM Strength			
	a_4*b_2	1.112	-0.125, 3.422
	a_5*b_2	1.246	-0.130, 3.777
	a_6*b_2	0.591	-0.714, 2.200
Mediators: Δ Muscle Thickness and 1RM Strength in Series			
	a_1*d*b_2	0.309	-0.058, 1.233
	a_2*d*b_2	0.506	-0.105, 1.890
	a_3*d*b_2	0.613	-0.128, 2.253

Estimated regression coefficients for the serial mediation model (Muscular Endurance). $a_{1\text{-}3}$ = adjusted mean difference in muscle thickness (Δ muscle thickness) between each training group and Control, $a_{4\text{-}6}$ = adjusted mean difference in 1RM strength (Δ 1RM strength) between each training group and Control, $c'_{1\text{-}3}$ = adjusted relative direct effect of each training group compared to Control, b_1 = adjusted effect of changes in muscle thickness on changes in muscular endurance, b_2 = adjusted effect of changes in 1RM strength on changes in muscular endurance, d = adjusted effect of changes in muscle thickness on changes in 1RM strength, $a_{1\text{-}3}*b_1$ = adjusted relative indirect effect of each training group on changes in muscular endurance through changes in muscle thickness compared to Control, $a_{4\text{-}6}*b_2$ = adjusted relative indirect effect of each training group on changes in muscular endurance through changes in 1RM strength compared to Control, $a_{1\text{-}3}*d*b_2$ = adjusted relative indirect effect of each training group on changes in muscular endurance through changes in muscle thickness and changes in 1RM strength in series compared to Control. Sex and pre-training values of muscle thickness, 1RM strength, and muscular endurance were included as covariates.
